# Recycling and Resource Recovery from Polymers

**DOI:** 10.3390/polym14102020

**Published:** 2022-05-16

**Authors:** Sheila Devasahayam, Raman Singh, Vladimir Strezov

**Affiliations:** 1 Department of Chemical and Biological Engineering, Faculty of Science and Engineering, Monash University, Melbourne, VIC 3800, Australia; raman.singh@monash.edu; 2 Department of Earth and Environmental Sciences, Faculty of Science & Engineering, Macquarie University, Sydney, NSW 2109, Australia; vladimir.strezov@mq.edu.au

Over 100 million tonnes of waste plastics is projected to enter our environment by 2030. Current waste management practices for the end-of-life (EOL) plastics globally are land filling, industrial energy recovery from municipal solid waste incineration (MSWI), pyrolysis and recycling, as shown in the figure [1]. Since 1950, only 9 precent of the plastics have been recycled, 19% incinerated and almost 50% went to sanitary landfill. Environmental challenges posed by wrong EOL plastic management drive the plastics recycling schemes for energy recovery, cutting emissions, penalties, energy consumption, non-renewable resources, and manufacturing costs.

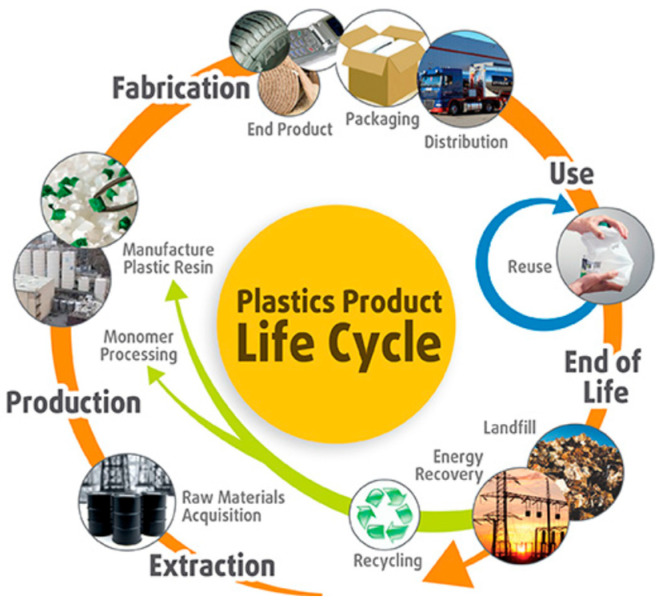



Life Cycle of Plastics.

Plastic recycling industry relies on the competitive cost and quality of recycled resins when compared with virgin materials and the added environmental footprint reduction/benefits. Recycling reportedly has the lowest environmental impact on both global warming potential and total energy use in most cases. However, underutilised plastic wastes due to low value issues with sorting/contamination pose major challenges.

Identification of novel technologies to drive innovation in a circular economy model for plastic can greatly help reduce plastic wastes. Circular economy model employs reuse, recycling and responsible manufacture solutions, support the development of new industries and jobs, reduce emissions and increase efficient use of natural resources (including energy, water and materials). Many economies are working towards achieving a zero plastic waste economy.

Plastics have a high calorific value when compared with other materials, making them a convenient energy and fuel source.

During the chemical feedstock recycling, plastics decompose without combustion, producing useful materials and syngas.

This special issue covers the applications of recycled plastics in the areas of energy recovery/alternative fuels, economic analyses, bitumen additives, flame retardants, recycled polymer nanocomposites to enhance the mechanical property, thermomechanical recycling to improve physical properties, mechano-chemical treatment, cryogenic waste tyre recycling, application in decarbonizing technology, e.g., cement industry, waste characterization, improving agricultural soil quality, as smart fertilizers, etc.

The Editors express their appreciation to all the contributors across the world in the development of this book. This book gives different perspectives and technical ideas for transformation of plastic wastes into value-added products and achieve higher recycling rates in the coming years.
